# Supplementary material for: Enhancing the evaluation of pathogen transmission risk in a hospital by merging hand-hygiene compliance and contact data: a proof-of-concept study
Source: BMC Res Notes. 2015 Sep 10;8:426. doi: 10.1186/s13104-015-1409-0 (PMC4566487; doi:10.1186/s13104-015-1409-0)
Supplement: Supplementary file 1 — Additional file 1. Additional analysis including in particular the daily contact patterns of healthcare workers and a detailed investigation of their hand-hygiene behaviour. [file 13104_2015_1409_MOESM1_ESM.pdf]

Enhancing the evaluation of pathogen transmission risk in a hospital by merging hand-hygiene compliance and contact data: a proof-of-concept study.

Additional file

Rossana Mastrandrea[1], Alberto Soto-Aladro[2,3], Philippe Brouqui[2,3], and Alain Barrat[1,4]

[1]Aix Marseille Université, Université de Toulon, CNRS, CPT, UMR 7332, 13288 Marseille, France

[2]Aix Marseille Université, URMITE, UM63, CNRS 7278, IRD 198, Inserm 1095, Marseille, France

[3]Infectious Disease Unit CHU Nord, Institut Hospitalo-Universitaire Méditerranée Infection, Marseille, France

[4]Data Science Laboratory, ISI Foundation, Torino, Italy

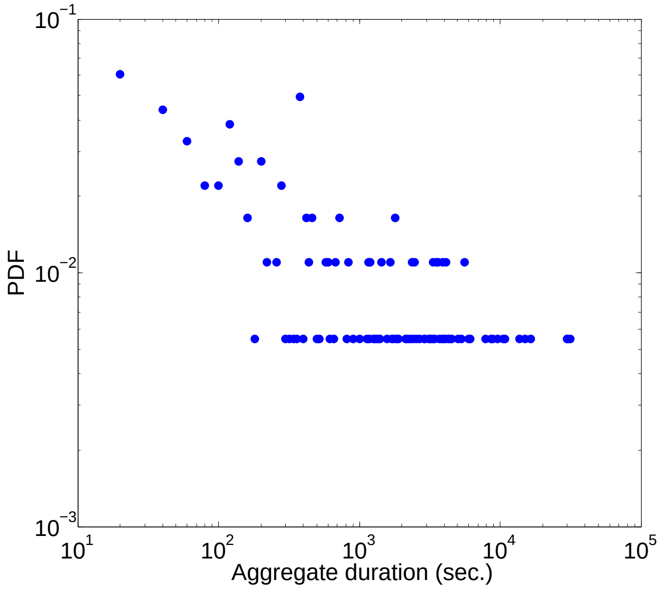

Figure S1: **Distribution of aggregate durations of contacts.** Distribution of the aggregated durations of contacts between HCWs, computed over the whole data collection.

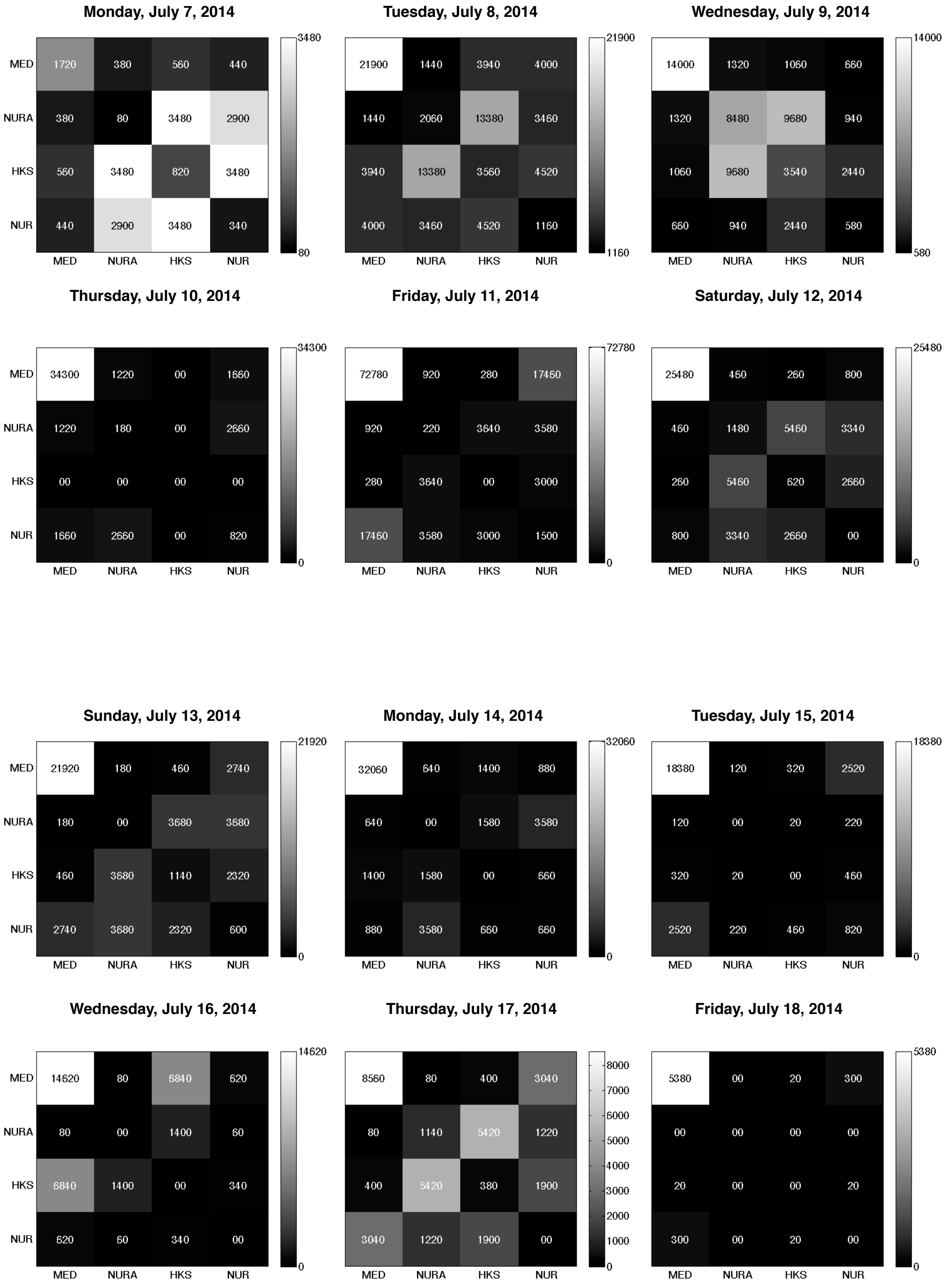

Figure S2: **Daily contact matrices.** Each matrix gives at row X and column Y the aggregate duration of contacts between HCWs of category X and HCWs of category Y during a specific day. Abbreviations: MED, medical doctors; NURA, nurses' aids; HKS, housekeeping staff; NUR, nurses.

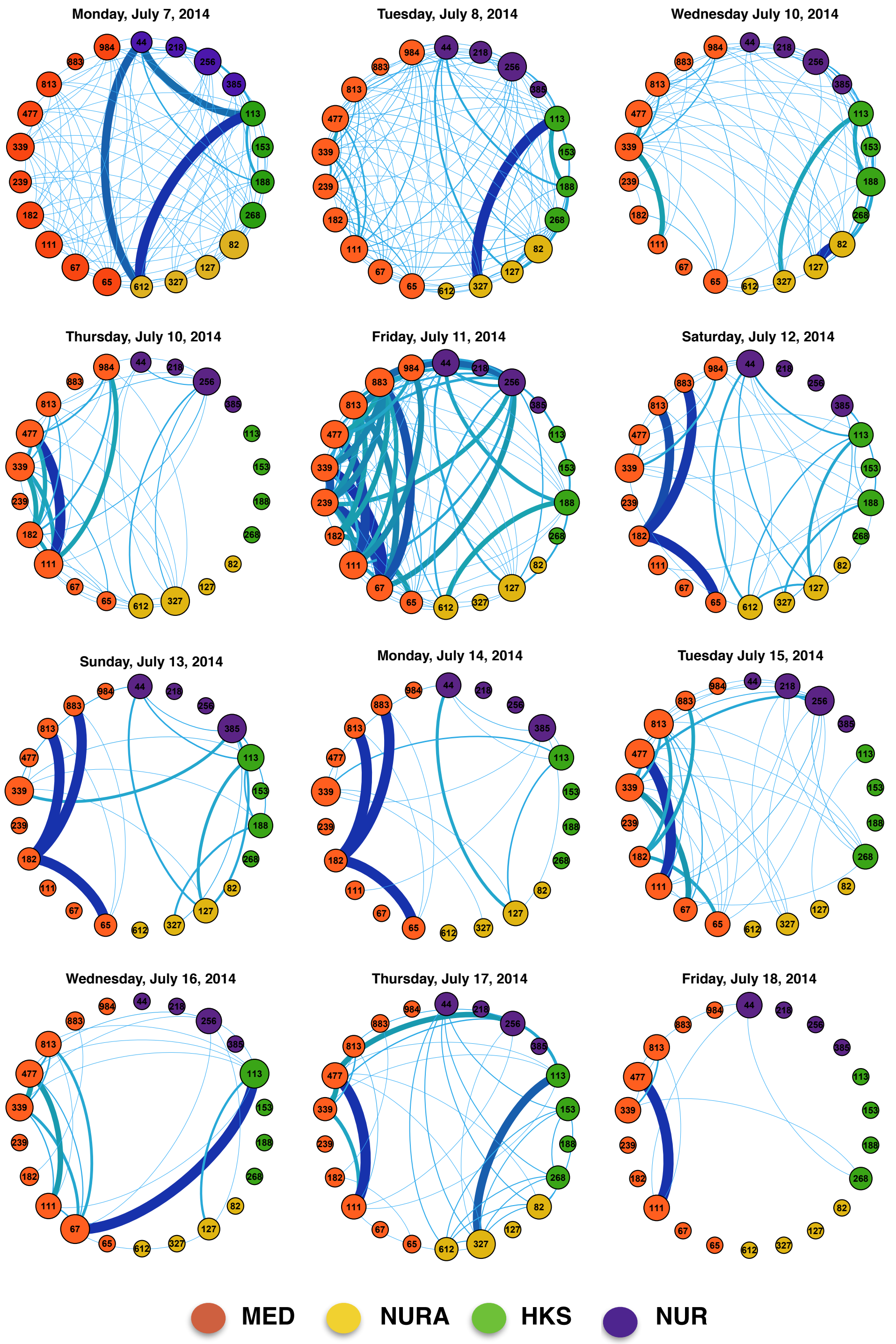

Figure S3: **Daily contact networks of health-care workers.** Visualization of the contact network of HCWs, aggregated at a daily scale. Each node represents a HCW (the number stands for the tag number, the color for the category as indicated in the color code). A link between two nodes exists if at least one contact between the corresponding HCWs was detected, and the link thickness gives the aggregate duration of contacts during the considered day. The size of each node is given by its degree (number of links). Abbreviations: MED, medical doctors; NURA, nurses' aids; HKS, housekeeping staff; NUR, nurses.

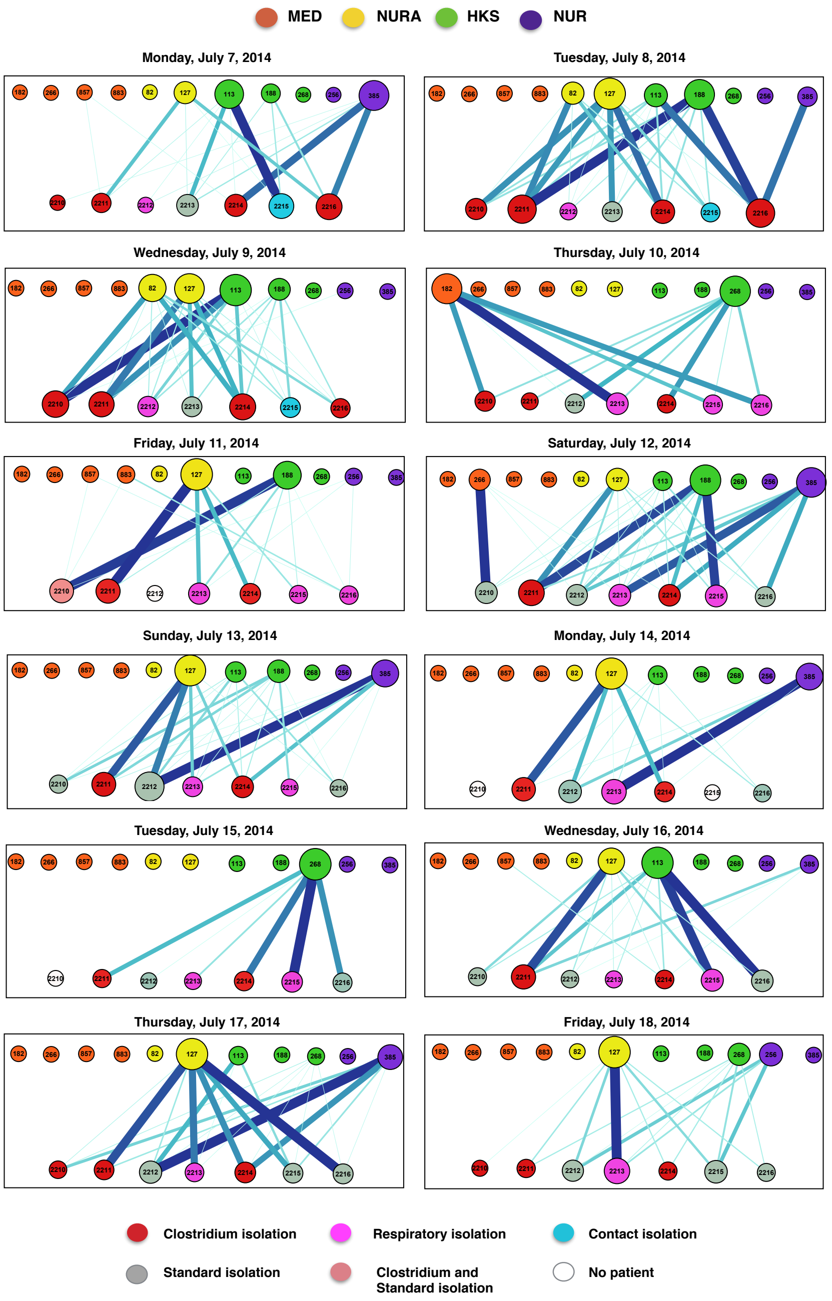

Figure S4: **Daily bipartite networks of contacts between health-care workers and patients.** Bipartite network of visits of HCWs to patient rooms. Each node represents either a HCW (top rows of each network) or a room (bottom rows). A link is drawn between a HCW and a room if the HCW visited the room at least once, and the link thickness depends on the aggregate duration of the visits. The size of a node depends on its strength (aggregate duration of visits). The color of each HCW node indicates its category, the color of each room node represents the type of isolation. Abbreviations: MED, medical doctors; NURA, nurses' aids; HKS, housekeeping staff; NUR, nurses.

MHT data	
Total visits	510 (321)
Visits without contact with patient	189 (37%)
Visits with contact and no use of HAS	156 (48.5%) (30.5%)
Visits with contact and use of HAS only BEFORE	26 (8%) (5%)
Visits with contact and use of HAS only AFTER	95 (29.5%) (19%)
Visits with contact and complete use of HAS	44 (14%) (8.5%)

(a)

	82 (NURA)	113 (HKS)	127 (NURA)	182 (MED)	188 (HKS)	256 (NUR)	266 (MED)	268 (HKS)	385 (NUR)	857 (MED)	883 (MED)
Visits with contact and no use of HAS	12 (50%)	22 (37%)	47 (47.5%)	0 (0%)	22 (42%)	0 (0%)	1 (50%)	11 (79%)	39 (76%)	2 (50%)	1 (50%)
Visits with contact and use of HAS only BEFORE	4 (17%)	7 (11.5%)	6 (6.5%)	0 (0%)	2 (4%)	0 (0%)	1 (50%)	1 (7%)	3 (6%)	1 (25%)	1 (50%)
Visits with contact and use of HAS only AFTER	7 (29%)	19 (31.5%)	31 (31%)	0 (0%)	23 (44%)	6 (67%)	0 (0%)	2 (14%)	7 (14%)	0 (0%)	0 (0%)
Visits with contact and complete use of HAS	1 (4%)	12 (20%)	15 (15%)	5 (100%)	5 (10%)	2 (23%)	0 (0%)	0 (0%)	2 (4%)	1 (25%)	0 (0%)
Total Visits	24 (100%)	60 (100%)	99 (100%)	5 (100%)	52 (100%)	8 (100%)	2 (100%)	14 (100%)	51 (100%)	4 (100%)	2 (100%)
Visits without contact	13	31	66	0	36	1	3	12	28	2	0

(b)

Figure S5: **Hand-hygiene compliance of HCWs**. (a) Total number of visits to patients in the whole period of data collection, divided according to the use of hydro-alcoholic solution before or/and after the contact with the patient. The numbers in blue are computed when taking into account only the visits that included a contact with the patient. (b) Number of visits to patients divided according to the use of HAS for each HCW monitored by the MHT system. The percentages are computed with respect to columns and do not take into account the visits without contacts with the patient. Abbreviations: MED, medical doctors; NURA, nurses' aids; HKS, housekeeping staff; NUR, nurses.

ISOLATION —————————— USE of HAS	STANDARD	CONTACT	RESPIRATORY	CLOSTRIDIUM	TOTAL
COMPLETE	11 (13%)	2 (14%)	12 (14%)	19 (14%)	44 (13%)
ONLY BEFORE	8 (10%)	3 (21.5%)	7 (8%)	8 (6%)	26 (8%)
ONLY AFTER	22 (26%)	3 (21.5%)	22 (27%)	48 (34%)	95 (30%)
NO USE	43 (51%)	6 (43%)	42 (51%)	65 (46%)	156 (49%)
TOTAL	84 (100%)	14 (100%)	83 (100%)	140 (100%)	321 (100%)

(a)

ISOLATION —————————— USE of HAS	STANDARD	CONTACT	RESPIRATORY	CLOSTRIDIUM	TOTAL
COMPLETE	4340 (14%)	560 (13%)	2580 (14%)	7660 (13%)	15140 (14%)
ONLY BEFORE	5520 (18%)	380 (9%)	1360 (7%)	1980 (3%)	9240 (8%)
ONLY AFTER	4100 (14%)	1280 (31%)	7460 (41%)	20220 (36%)	33060 (30%)
NO USE	16060 (54%)	1940 (47%)	6840 (38%)	27180 (48%)	52020 (48%)
TOTAL	30020 (100%)	4160 (100%)	18250 (100%)	57040 (100%)	109460 (100%)

(b)

Figure S6: **Hand-hygiene compliance of HCWs and type of room isolation**. (a) Number and (b) cumulative duration of visits to patients in the whole period of data collection, divided according to the use of hydro-alcoholic solution before and after the visit and the type of room isolation.
